# Acupuncture combined with metformin for polycystic ovary syndrome: A protocol for systematic review and meta-analysis

**DOI:** 10.1097/MD.0000000000032234

**Published:** 2022-12-09

**Authors:** Yanan Sun, Xiuping Liu, Qi Ding, Shanshan Yin, Hongyu Yang

**Affiliations:** a Department of Gynecology and Obstetrics, The 980th Hospital of the Joint Logistic Support Force of PLA, China; b Department of Gynecology and Obstetrics, The Third Hospital of Hebei Medical University, China.

**Keywords:** acupuncture, meta-analysis, metformin, polycystic ovary syndrome, systematic review

## Abstract

Polycystic ovary syndrome is an endocrine disorder that occurs mostly in women of reproductive age and has lifelong effects on their health.

**Methods:**

Randomized controlled clinical trials that met the inclusion criteria were screened by searching multiple databases. Relevant data were extracted, and meta-analysis was performed using Reviewer Manager 5.4.

**Results:**

The results of this study will be submitted to peer-reviewed journals.

**Conclusion:**

This study provides evidence for the efficacy and safety of the clinical application of acupuncture combined with metformin in the treatment of polycystic ovary syndrome.

## 1. Introduction

Polycystic ovary syndrome (PCOS) is a complex endocrine disorder that affects > 10% of women of childbearing age.^[1]^ PCOS often leads to androgen overproduction and infertility^[2,3]^ PCOS is often associated with overproduction of androgens, and is closely related to obesity, type 2 diabetes, insulin resistance, etc.^[4–6]^ It is also associated with obesity, type 2 diabetes, and insulin resistance. In one study, the risk of obesity was 4 times higher in PCOS patients than in the controls.^[7]^ In addition, PCOS increases the risk of endometrial cancer in women.^[8]^ For pregnant women, PCOS increases the risk of miscarriage, hypertension, and gestational hyperglycemia.^[9]^ PCOS seriously impacts women’s health and puts strain on the global health system.

The main current treatment modalities for PCOS and its comorbidities include lifestyle improvements, pharmacotherapy, and surgery. Current evidence suggests that lifestyle improvement can reduce body weight, free androgen index, and body mass index.^[10]^ In terms of pharmacological treatment, metformin combined with lifestyle improvements can alleviate insulin resistance and improve lipid distribution.^[11]^ Surgery should be considered when other treatment modalities are ineffective. However, the patient’s specific situation and risks of surgery should be fully considered.

Acupuncture, a millennial-old alternative therapy, is widely used in clinical practice because of its low risk, effectiveness, and high patient acceptance. A large amount of clinical evidence shows that acupuncture combined with drugs or applied alone has a positive effect on PCOS.^[12–14]^ However, the sample sizes included in these clinical trials were relatively small, and the specific effects of acupuncture in the treatment of PCOS cannot be accurately assessed.

In this systematic review and meta-analysis, the efficacy and safety of acupuncture therapy in the treatment of PCOS were investigated by comparing the data of acupuncture combined with metformin and metformin alone, or metformin combined with sham acupuncture, aiming to provide a scientific basis for the clinical application of acupuncture in the treatment of PCOS.

## 2. Materials and Methods

This protocol has been registered with the International Prospective Register of Systematic Reviews under the registration number CRD42022373019. It follows the Preferred Reporting Items of the Program for Systematic Evaluation and Meta-Analysis.

### 2.1. Types of studies

We will select randomized controlled clinical trials (RCTs) of acupuncture combined with metformin for the treatment of PCOS, without restriction of region or language.

### 2.2. Participants

Patients with a definite diagnosis of PCOS were included in this study. All eligible participants will be included, with no restrictions on race or nationality.

### 2.3. Intervention

The intervention in the experimental group was acupuncture combined with metformin without restricting the dose or form of metformin. Acupuncture includes electroacupuncture and hand acupuncture. The control group was treated with metformin or metformin combined with sham acupuncture, without limiting the dose or form of metformin.

### 2.4. Exclusion criteria

Reviews, animal studies, conference papers, case reports, duplicated literature, and studies using special acupuncture as an intervention method that did not meet the criteria will be excluded.

### 2.5. Types of outcome measures

#### 2.5.1. Main outcome

The main objective of this study was to investigate the efficacy and safety of acupuncture in PCOS; therefore, we selected the main outcome indicators as the total effective rate. Serum sex hormone levels (luteinizing hormone, follicle-stimulating hormone, testosterone, T).

#### 2.5.2. Secondary outcomes

Secondary indicators will include Anthropometric indicators (waist circumference, waist-to-hip ratio, and body mass index). Metabolic replacement markers of IR (Homeostasis Model Assessment index and insulin sensitivity index). Adverse events and Incidence.

### 2.6. Literature search

#### 2.6.1. Literature sources

We searched PubMed, Cochrane Library, Web of Science, Embase, MEDLINE, China Biology Medicine, Scopus, CNKI, VIP, and Wanfang databases. In addition, we will also search for references of the included literature, Open Grey, ClinicalTrials.gov registered clinical studies, and other relevant resources will also be searched. For ongoing or unpublished RCTs, the authors will be contacted by email and other means to obtain relevant experimental data. The time frame for the search was from build to November 4, 2022.

#### 2.6.2. Search strategy

We will use “PCOS” “Stein-Leventhal Syndrome” “Sclerocystic Ovarian Degeneration” “Sclerocystic Ovary Syndrome” “Metformin” “ Dimethylbiguanidine” “Metformin Hydrochloride” “Metformin HCl” “Acupuncture” “Pharmacopuncture” “electroacupuncture” “randomized controlled trial” “randomized” “placebo” etc were used as keywords for the search. The specific search strategy is presented in Table 1 (PubMed was used as an example).

**Table 1:** Search strategy used in PubMed database.

Number	Search terms
#1	“Polycystic Ovary Syndrome”[Mesh]
#2	Ovary Syndrome, Polycystic[Title/Abstract]
#3	Syndrome, Polycystic Ovary[Title/Abstract]
#4	Stein-Leventhal Syndrome[Title/Abstract]
#5	Stein Leventhal Syndrome[Title/Abstract]
#6	Syndrome, Stein-Leventhal[Title/Abstract]
#7	Sclerocystic Ovarian Degeneration[Title/Abstract]
#8	Ovarian Degeneration, Sclerocystic[Title/Abstract]
#9	Sclerocystic Ovary Syndrome[Title/Abstract]
#10	Polycystic Ovarian Syndrome[Title/Abstract]
#11	Ovarian Syndrome, Polycystic[Title/Abstract]
#12	Polycystic Ovary Syndrome 1[Title/Abstract]
#13	Sclerocystic Ovaries[Title/Abstract]
#14	Ovary, Sclerocystic[Title/Abstract]
#15	Sclerocystic Ovary[Title/Abstract]
#16	#1 or #2 or #3 or #4 or #5 or #6 or #7 or #8 or #9 or #10 or #11 or #12 or #13 or #14 or #15
#17	“Acupuncture Therapy”[Mesh]
#18	Acupuncture Treatment[Title/Abstract]
#19	Acupuncture Treatments[Title/Abstract]
#20	Treatment, Acupuncture[Title/Abstract]
#21	Therapy, Acupuncture[Title/Abstract]
#22	#17 or #18 or #19 or #20 or #21
#23	“Metformin”[Mesh]
#24	Dimethylbiguanidine[Title/Abstract]
#25	Dimethylguanylguanidine[Title/Abstract]
#26	Glucophage[Title/Abstract]
#27	Metformin Hydrochloride[Title/Abstract]
#28	Hydrochloride, Metformin[Title/Abstract]
#29	Metformin HCl[Title/Abstract]
#30	HCl, Metformin[Title/Abstract]
#31	#23 or #24 or #25 or #26 or #27 or #28 or #29 or #30
#32	randomized controlled trial[Publication Type] or randomized[Title/Abstract] or placebo[Title/Abstract]
#33	#16 and #22 and #31 and #32

### 2.7. Literature collection and organization

**Figure 1.**
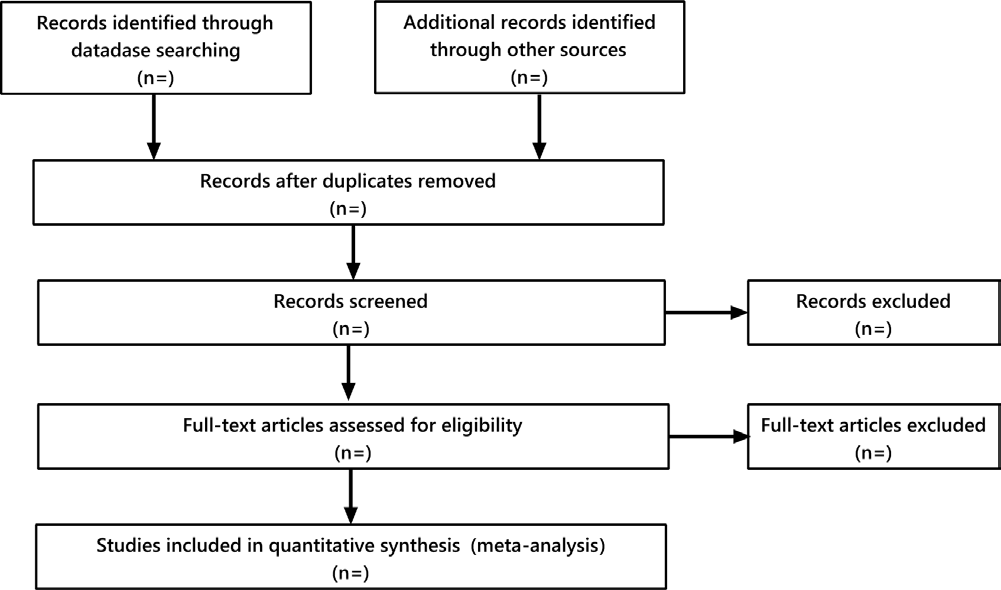
The process of literatures screening.

Two researchers performed the data collection and collation separately. The literature that met the criteria was first managed by applying EndNote, and duplicates were removed. Two researchers screened the literature by reading the titles, abstracts, and keywords, and the reasons for exclusion were recorded for the excluded literature. In case of disagreement between the 2 researchers, a third researcher will make the decision. The detailed screening process is illustrated in Figure 1.

### 2.8. Data extraction and export

Two researchers extracted the data from the screened literature and created an Excel spreadsheet. Valid information extracted included the title, author, year, diagnostic criteria, sample size, age, duration of disease, intervention modality, treatment period, outcome indicators, follow-up time, and adverse events. In case of disagreement in this process, it was resolved by discussion between 2 investigators, and a third investigator was invited to participate in the evaluation if necessary. If the data in the literature were inadequate or inaccurate, one investigator contacted the author of the literature to seek more information.

### 2.9. Risk of bias analysis of the included literature

To assess the methodological quality of the included studies, the risk of bias was assessed by 2 researchers using the Cochrane tool for the included literature. Each study was classified as having high, low, or unclear risk according to the following 7 items: random sequence generation, allocation concealment, blinding of participants and personnel, blinding of outcome assessment, incomplete outcome data, selective reporting, and other biases. This process was decided by a third investigator if a disagreement arose.

### 2.10. Data synthesis

A meta-analysis was performed by 2 researchers who used Reviewer Manager 5.4. The 95% confidence intervals were used, mean differences were calculated for continuous variables, and risk ratios were calculated for dichotomous variables. Data heterogeneity was assessed using the chi-square and *I*^2^ tests. When heterogeneity was not significant (*P *≥ .10, *I*^2^ ≤ 50%), a fixed-effects model was used for analysis; when heterogeneity was significant (*I*^2^ > 50% or *P *< .10), a random-effects model was used.

### 2.11. Heterogeneity assessment

Data heterogeneity was assessed using the chi-square and *I*^2^ tests. When heterogeneity was not significant (*P* ≥ .10, *I*^2^ ≤ 50%), a fixed-effects model was used for the analysis; when heterogeneity was significant (*I*^2^ > 50% or *P* < .10), a random-effects model was used.

### 2.12. Assessment of reporting biases

Reporting bias assessments will be performed when necessary to ensure the accuracy of the study results. If >10 publications were included, the symmetry of the funnel plots was assessed using Stata 14.0.

### 2.13. Subgroups analysis

If there was significant heterogeneity in the results of the data analysis, we performed a subgroup analysis. This will be explored according to age, race, treatment period, sample size, or other factors that may affect the results.

### 2.14. Sensitivity analysis

To ensure the reliability of the conclusions, we performed a sensitivity analysis based on method quality, sample size, and missing data. Data analysis and comparison of the results will be performed to assess the reliability of the results.

### 2.15. Ethics and dissemination

The data in this study were obtained from the published literature and therefore did not require ethical approval. The results of this study will be submitted to peer-reviewed journals.

## 3. Discussion

A large body of clinical evidence confirms the clinical efficacy of acupuncture in combination with metformin in PCOS; however, there is a lack of evidence-based medical support for this regimen in the treatment of PCOS. In this study, the efficacy and safety of acupuncture combined with metformin in the treatment of PCOS were investigated by analyzing data from RCTs of this treatment modality. The results of this study will provide a scientific basis for the clinical application of acupuncture combined with metformin in the treatment of PCOS and facilitate the optimization of treatment protocols for this disease.

## Author contributions

Yanan Sun and Xiuping Liu contributed equally to the work and should be regarded as co-first authors. All the authors agree with the publication of the protocol.

**Conceptualization:** Yanan Sun, Hongyu Yang.

**Data curation:** Xiuping Liu, Qi Ding.

**Formal analysis:** Xiuping Liu.

**Methodology:** Qi Ding, Shanshan Yin.

**Software:** Yanan Sun, Xiuping Liu.

**Supervision:** Hongyu Yang.

**Writing – original draft:** Yanan Sun, Xiuping Liu.

**Writing – review & editing:** Hongyu Yang.
